# Chemical ecology of symbioses in cycads, an ancient plant lineage

**DOI:** 10.1111/nph.70109

**Published:** 2025-03-28

**Authors:** Shayla Salzman, Edder D. Bustos‐Díaz, Melissa R. L. Whitaker, Adriel M. Sierra, Angélica Cibrián‐Jaramillo, Francisco Barona‐Gómez, Juan Carlos Villarreal Aguilar

**Affiliations:** ^1^ Department of Entomology University of Georgia Athens GA 30602 USA; ^2^ Naturalis Biodiversity Center Darwinweg 2 2333 CR Leiden the Netherlands; ^3^ Biological Sciences East Tennessee State University Johnson City TN 37614 USA; ^4^ Département de Biologie Université Laval G1V 0A6 Québec City Quebec Canada; ^5^ Evolution of Microbial Chemodiversity Laboratory Institute of Biology, Leiden University 2333 BE Leiden the Netherlands; ^6^ Smithsonian Tropical Research Institute (STRI) Ancón Panama

**Keywords:** chemical ecology, cyanobacteria, cycads, gymnosperms, microbiome metabolites, plant secondary metabolites, symbiosis, volatile organic compounds

## Abstract

Cycads are an ancient lineage of gymnosperms that maintain a plethora of symbiotic associations from across the tree of life. They have myriad morphological, structural, physiological, chemical, and behavioral adaptations that position them as a unique system to study the evolution, ecology, and mechanism of symbiosis. To this end, we have provided an overview of cycad symbiosis biology covering insects, bacteria, and fungi, and discuss the most recent advances in the underlying chemical ecology of these associations.

## Resumen

Las cícadas son un antiguo linaje de gimnospermas que contienen una plétora de asociaciones simbióticas con organismos de todo el árbol de la vida. Ellas presentan una miríada de adaptaciones morfológicas, estructurales, fisiológicas, químicas y de comportamiento que las sitúan como un sistema único para estudiar la evolución, la ecología y el mecanismo de simbiosis. Con este fin, hemos proporcionado una visión general de la biología de la simbiosis de las cícadas que abarca insectos, bacterias y hongos, y discutimos los avances más recientes en la ecología química relacionada a estas asociaciones.

## Introduction

**Fig. 1:**
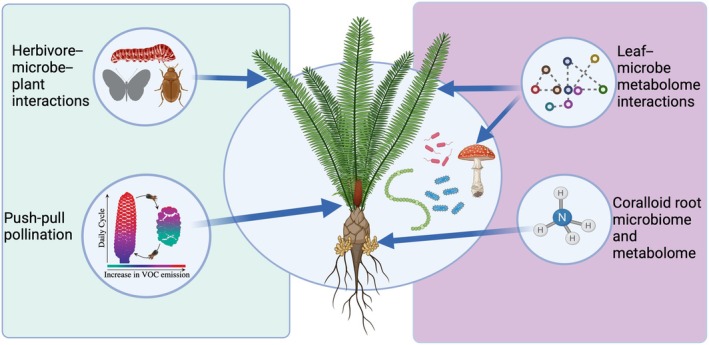
Conceptual summary of cycad chemical ecology research presented in this review. Obligate brood‐site pollinators are manipulated by cyclical changes in cone scent chemistry (volatile organic compound (VOC)) that drive a push‐pull pollination mechanism that has existed since at least the mid‐Jurassic. Cycads have highly specialized herbivores, mostly butterfly caterpillars that display aposematic coloring and have, in some cases, been shown to sequester plant secondary metabolites such as methylazoxymethanol glycosides or β‐methylamino‐l‐alanine. Morphologically distinct secondary roots harbor a cyanobacterial‐driven consortium that provides nitrogen (ammonia) to the plant and produces a host of chemical compounds. Most recent research has provided a novel insight on the interaction between the leaf microbiota (fungi and bacteria) and the metabolites produced by the plant and as a product of the interaction. Figure created with Biorender.com (https://BioRender.com/n44v963).

Cycad biologist Knut Norstog once suggested that we should use ‘the analogy of the Rosetta Stone for the fund of information stored within the living cycads and its importance to the interpretation of plant biology….the very ancient structures and developmental pathways of cycads enables us to make connections between the early origins of seed plants and their present‐day counterparts’ (Donaldson, 2003). Indeed, cycads maintain a plethora of traits that have influenced our understanding of land plant evolution, the origin of insect pollination, symbiosis biology, and coevolution (Norstog & Nicholls, 1997). Chemical ecology is integral to many of these traits, such as thermogenesis in reproductive tissues, symbiotic brood‐site pollination, specialized insect associates with diverse defensive ecologies, and morphologically distinct coralloid roots for housing nitrogen‐fixing microbiota. For centuries, cycad research has touched every corner of plant biology including ecology, evolution, physiology, ethnobotany, phylogenetics, systematics, development, genomics, signaling, and paleobotany. Due to recent methodological and technical advances, cycad research is now, more than ever, uniquely positioned to address basic questions across the biological sciences. Here, we synthesize the current state of cycad chemical ecology and present the most recent advances in cycad research on the evolution and mechanisms of insect pollination and herbivory, the biochemical basis of symbiosis, and microbial symbionts (Fig. 1).

## Cycad biology

Cycads are one of the largest extant gymnosperm lineages, comprising 10 genera and *c*. 375 species, two‐thirds of which are included on the International Union for Conservation of Nature (IUCN) Red List of threatened species (Calonje *et al*., 2023). Cycads are among the most ancient extant seed plants, with likely origins in the Carboniferous. They are currently globally distributed in the tropics and subtropics, where they hold considerable cultural significance, with a rich history of anthropological and ethnobotanical research (e.g. Carrasco *et al*., 2022).

**Fig. 2:**
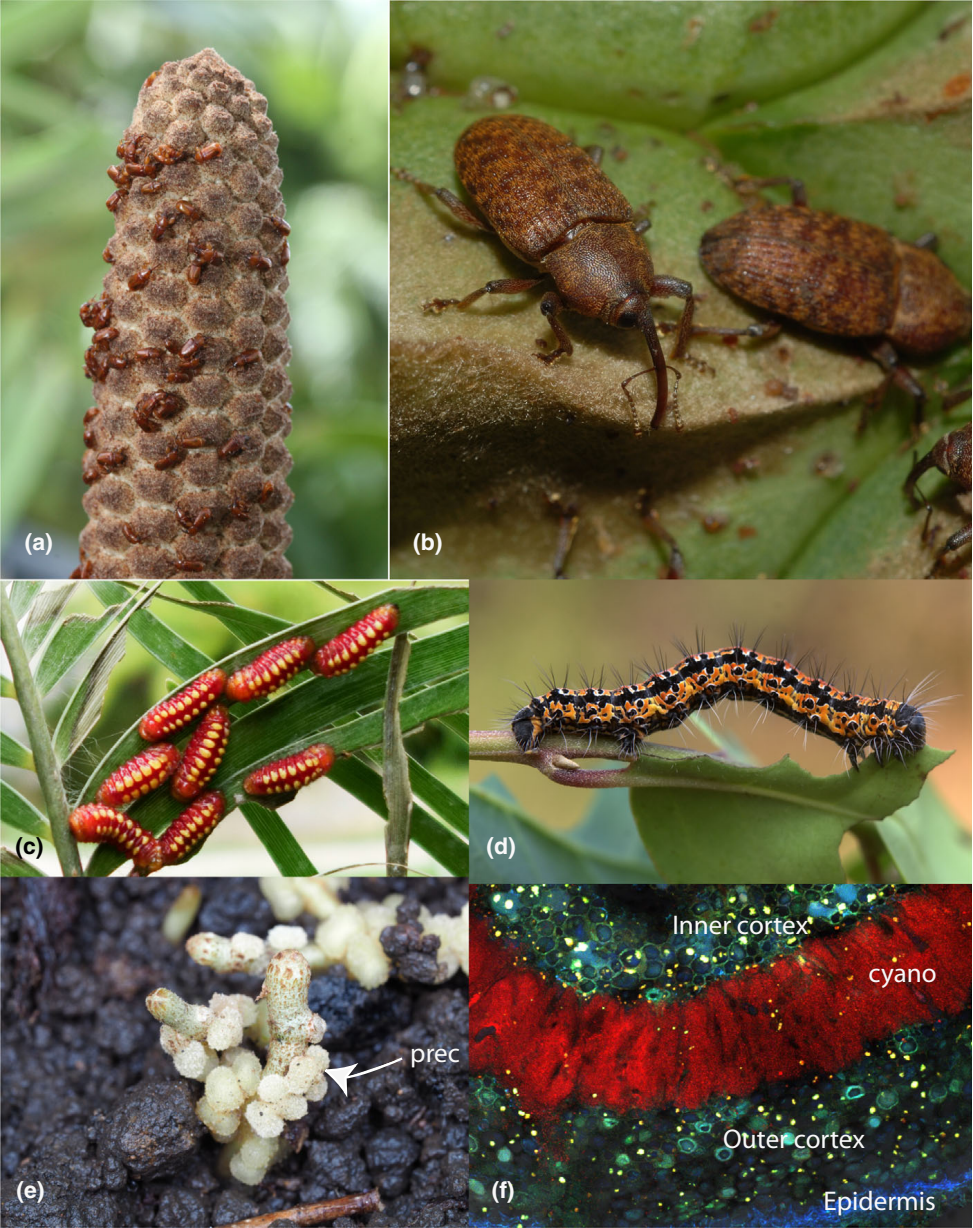
Examples of cycad mutualistic interactions mediated by chemistry. (a). *Zamia furfuracea* pollen cone with *Rhopalotria furfuracea* weevil pollinators. (b). *Tranes lyterioides* weevil pollinators of *Lepidozamia peroffskyana*. (c). Gregarious obligate cycad‐feeding *Eumaeus atala* butterfly larvae show bright red aposematic coloring. (d). *Zerenopsis lepida* moth larvae are obligate cycad feeders in their first instar and may subsequently shift hosts, yet are also aposematically colored. (e). Young pre‐coralloid roots (prec) in *Zamia nana*. (f). Fluorescence microscopy of a coralloid root showing the cyanobacterial zone (cyano in red) between the inner and outer cortex. Photo credits: Michael Calonje, Nicholas Fisher, Rolf Oberprieler, Shayla Salzman, Janse van Rensburg *et al*., 2023, M. Madrid, and J. Ceballos.

Cycads exhibit a striking amount of mutualism in their associations with insects. Entire lineages of both herbivores and pollinators are specialized on cycads, in what has been suggested to be classic examples of coevolution (Tang *et al*., 2020; Whitaker & Salzman, 2020). All of these insects must contend with a host of cycad secondary metabolites, some of which are rather rare in the known biological world, and many of the specialist insects are aposematically colored (Fig. 2c,d; Whitaker & Salzman, 2020). These dioecious gymnosperms appear to rely almost exclusively on insect vectors for pollination, which they maneuver between cone sexes through a series of physiological events that include cone thermogenesis (Terry *et al*., 2016). The brood‐site pollination mutualists live their entire life cycles within the reproductive structures of their host cycad, feeding, breeding, and laying eggs within the tissue (Terry *et al*., 2012).

Cycads also engage in symbiosis with microbiota. The plants produce morphologically distinct coralloid roots that house fungi, nitrogen‐fixing cyanobacteria, and associated bacteria (Zheng *et al*., 2018; Suárez‐Moo *et al*., 2019; Bell‐Doyon *et al*., 2020), the functions of which are only recently being elucidated (Gutiérrez‐García *et al*., 2019). More recent research suggests that leaf microbial associates may also contribute to plant growth success and habitat diversity, including nitrogen fixation in the world's only epiphytic gymnosperm species (*Zamia pseudoparasitica*: Sierra *et al*., 2024).

## Pollination and herbivory

Like other land plants, cycads' interactions with animals (mostly insects) include parasitic and mutualistic relationships that are largely mediated by the plants' chemical and morphological characteristics. But cycads' deep evolutionary history, thermogenesis, unique chemistries, and defensive traits make them somewhat unusual in the context of plant–insect interactions. These traits position cycads as especially promising for understanding a suite of ‘big questions’ in plant–insect interactions, relating to the evolutionary origins of insect pollination and phytophagy, the origins and maintenance of phytochemical diversity, insect counter‐adaptations to plant defenses, and plant signaling and communication.

### Ancient pollination and multi‐modal partner‐encounter signals

Fossil and phylogenetic evidence suggests cycads have maintained obligate insect pollination since before the rise of flowering plants (Cai *et al*., 2018; Salzman *et al*., 2020). While cycads were historically thought to be exclusively wind‐pollinated, brood‐site pollination mutualists have now been found in most cycad species, overwhelmingly involving Coleoptera (beetles and weevils), but sometimes Thysanoptera (thrips) or Lepidoptera (moths) (Tang, 2004; Terry *et al*., 2012; Salzman *et al*., 2020). These brood‐site pollinators feed, breed, and develop on plant reproductive tissue – almost exclusively on pollen cones, although one obligate ovule parasite may play a small role in the pollination of at least one species (Donaldson, 1997).

The dioecious nature of these plants that rely on pollinators that live within only one of their cone sexes means that cycads must utilize partner‐encounter signaling to attract their pollinators and yet must also manage their movement between cone sexes of a nearby conspecific to complete pollination. This is thought to be chemically mediated by changes in cone volatile organic compounds (VOCs). In both thrips pollinators of *Macrozamia* and weevil pollinators of *Zamia*, this occurs through a ‘push‐pull’ pollination process whereby pollinators are attracted to lower quantities of cone VOCs and repelled by high VOC quantities (Terry *et al*., 2007b; Salzman *et al*., 2020, 2021). Pollen and ovulate cones across cycads undergo a daily process of increased respiration and thermogenesis that is followed closely by an increase in cone VOCs (Terry *et al*., 2016; reviewed in Salzman *et al*., 2020). Comparative cone VOC analyses across cycads have shown that ovulate cones mimic pollen cone scent and emission patterns but have much lower emission rates (Terry *et al*., 2012). In the *Macrozamia* and *Zamia* systems studied, the ovulate cones have been shown to be attractive at peak VOC emission times when pollen cones become repellent. This has been hypothesized to be a form of ‘pollination by mistake’ whereby pollinators are tricked into visiting the ovulate cone where they would not otherwise aggregate in large numbers (Tang, 2004; Salzman *et al*., 2020). This push‐pull pollination appears to be ancestral in the lineage (Salzman *et al*., 2020), even if not explicit in all species (Suinyuy *et al*., 2013) and fossil evidence dates Coleopteran‐cycad brood site pollination to at least 167 million years ago (Cai *et al*., 2018) and perhaps as early as the Triassic (Kalvins *et al*., 2005), placing this pollination mechanism before the rise of flowering plants and as the oldest pollination mechanism yet documented (Salzman *et al*., 2020). The likely antiquity of this pollination strategy makes cycad pollination a rich case study on the mechanisms and maintenance of plant–insect mutualisms.

Chemical communication with pollination mutualists appears to be evolving across lineages and even within populations of species, as would be expected from coevolving or co‐speciating lineages. In the Caribbean clade of *Zamia*, chemical phenotypes were found to be more divergent between species than morphometric phenotypes (Salzman *et al*., 2021). The same study found a trend for higher rates of positive selection in VOC‐associated genes than in other genes, but the methods used to characterize gene function identified small numbers of ‘VOC genes’, leading to a lack of significance owed to small gene sample sizes. However, work within *Encephalartos* has shown divergence in both cone VOCs and pollinator preferences across populations of at least two different species (Suinyuy *et al*., 2015; Suinyuy & Johnson, 2018, 2021) further suggesting an evolutionary pressure for phenotype matching.

While a cone can produce a handful of chemical compounds, pollinating insects may only be physiologically capable of perceiving, and thus behaviorally responsive to, a reduced set of compounds. Identifying these ecologically active compounds involves physiological electroantennograph and behavioral choice assays using live insects. This has only been tested in five cycad species but presents some interesting patterns that further suggest the evolution of communication phenotypes. Two species of *Zamia* that are pollinated by two different species of *Rhopalotria* weevils produce very structurally different chemical compounds that influence their pollinator's behavior, the linear hydrocarbon 1,3‐octadiene and the benzoate ester methyl salicylate (Salzman *et al*., 2020, 2021). Within *Macrozamia*, *Cycaothrips chadwhiki* thrips and *Tranes* sp. weevil pollinators of *M. lucida* and *M. machinii* respectively both perceive B‐myrcene produced by their host plants, but diverge in the perception of additional volatiles: (E)‐B and (allo‐) ocimine elicits a response in *C. chadwhicki* while linalool does so in *Tranes* sp. but not vice versa (Terry *et al*., 2007a,b). It is also noteworthy that of the eight compounds that have been found to produce a physiological response in pollinators, 1,3‐octadiene is active in both *Zamia* and *Encephalartos*, yet relatively rare in the rest of Plantae. Further investigations in ecologically active cycad VOCs are necessary to look for evidence of private channels of communication between partners.

Cycads also provide an excellent system to investigate mechanisms of partner‐encounter signaling with implications for understanding early insect pollination before the overt visual signaling of flowers. The metabolic process of thermogenesis produces many potential cues (temperature, carbon dioxide (CO_2_), and humidity) that insects are innately capable of perceiving and were likely used for host plant localization before, and in conjunction with, the evolution of visual or odor signals (Peris *et al*., 2024). Indeed, cycad cone humidity has been shown to affect pollinator behavior (Terry *et al*., 2014; Salzman *et al*., 2023) and has recently been suggested to perform a signal‐like function in pollination in general (Dahake *et al*., 2022; Salzman *et al*., 2023). It is becoming increasingly clear that studies of plant signaling should be extended beyond visual and chemical signals to include humidity (von Arx *et al*., 2012; Terry *et al*., 2014; Dahake *et al*., 2022; Salzman *et al*., 2023), temperature (Seymour & Matthews, 2006; Terry *et al*., 2014), and CO_2_ (Goyret *et al*., 2008). Given the evolutionary history, pollination mutualisms, and variety of plant signals, research on cycad pollination mutualisms has the potential to provide new insight into the evolution of insect pollination through the lens of partner‐encounter and multi‐modal signaling.

### Parasitic insect herbivores

Cycads' unique chemistries are also important for understanding their associations with parasitic insect herbivores. Cycads produce potent phytotoxins that are unique among land plants, such as methylazoxymethanol (MAM) and β‐methylamino‐l‐alanine (BMAA), and lesser studied compounds such as steryl glycosides, with potentially more still to be discovered. For instance, the recently sequenced genome of *Cycas panzhihuaensis* contains a gene, *fitD*, that is thought to confer additional insecticidal properties (Liu *et al*., 2022). Likely acquired through horizontal gene transfer from a fungal microbe, the *Cycas fitD* gene encodes proteins that are similar to insecticidal toxins produced by *Photorhabdus* bacteria, and injection of the synthesized *Cycas fitD* protein into the larvae of two noncycadivorous moth species significantly increased larval mortality relative to controls, suggesting the protein confers protection from at least some insect herbivores.

Despite cycads' rich arsenal of chemical defenses, cycad‐feeding has been documented in at least six insect orders, with most cycad herbivores belonging to the orders Lepidoptera (moths and butterflies), Coleoptera (beetles and weevils), and Hemiptera (true bugs). A previous review of cycad‐feeding Lepidoptera concluded that cycad‐feeding has evolved independently multiple times among moths and butterflies, and that the defensive traits of cycad‐feeding lepidopteran lineages may be especially important in determining which lineages diversify (Whitaker & Salzman, 2020). Many cycad‐feeding coleopterans provide pollination services to cycads, but there are plenty of strictly parasitic cycad herbivores within the Coleoptera as well, including in the families Curculionidae (Hsiao & Oberprieler, 2020) and Cerambycidae (Marler, 2013), and subfamilies Aulacoscelinae (Prado *et al*., 2011) and Criocerinae (Wilson, 2021) as well as the Languriidae tribe of Erotylidae (Windsor *et al*., 1999). These groups display an incredible diversity of feeding ecologies, species interactions, and defensive traits, such that a systematic review of cycad‐feeding Coleoptera would be highly beneficial for synthesizing broader ecological and evolutionary patterns of cycad herbivory.

Many cycad‐associated insects are obligate cycad specialists and must possess adaptations to contend with cycads' chemical defenses. In general, herbivorous insects can excrete, sequester, or detoxify phytotoxins, but it is not clear which strategies are used by most cycad herbivores. There is some evidence that pollinating weevils excrete BMAA in their frass and pupal casings (Norstog & Fawcett, 1989), and some cycad‐feeding Aulacoscelinae beetles reflexively bleed MAM‐glycosides – presumably sequestered from their food plants – when disturbed or threatened (Prado *et al*., 2011). Given that many cycad‐feeding herbivores are aposematically colored (Fig. 2c,d as examples), it is presumed that the sequestration of cycad toxins confers a protective advantage to aposematic herbivores. At least one aposematic lepidopteran, *Seirarctia echo*, can detoxify MAM via glycosylation and accumulate cycasin, a nontoxic MAM‐glycoside, in its larval tissues (Teas *et al*., 1966; Teas, 1967). Cycasin was also reported in the adult tissues of museum specimens of several cycadivorous butterfly species (*Eumaeus minyas*, *Luthrodes cleotas*, *Taenaris butleri*, *Taenaris catops*, and *Taenaris onolaus*) by Nash *et al*. (1992) . Relatively few studies have investigated BMAA sequestration by cycad herbivores, and the potential defensive value of sequestering BMAA for protection against higher trophic levels is questionable given the compound's latent toxicity to most animals (Whitaker *et al*., 2022). The obligate cycad herbivore, *Eumaeus atala*, was found to accumulate BMAA in its larval and adult tissues, but in quantities that did not deter feeding by an invertebrate predator (Fig. 2c; Whitaker *et al*., 2023). Genomic evidence does suggest, however, that toxin tolerance is a key adaptation in the radiation of *Eumaeus* butterflies, a wholly cycadivorous neotropical genus of six species (Robbins *et al*., 2021). Finally, it has also been suggested that insects' gut microbiomes may contribute to degrading cycad toxins (Salzman *et al*., 2018), potentially via novel siderophore activity (Gutierrez‐Garcia *et al.*, 2023), though this remains to be experimentally demonstrated.

Just as obligate cycad pollinators must possess adaptations to locate their hosts, so must specialized cycad herbivores, but very little is known about the chemical, thermal, or visual cues used in the selection of feeding and oviposition sites. The field would benefit from future investigation into the underpinnings of host selection among cycad herbivores, as comparing the cues used by parasitic herbivores vs beneficial pollinators would provide a clearer view of the potential selective pressures that insects exert on cycad signals, especially chemical signals. Furthermore, a more mechanistic view of host selection would help assess the potential for host switching as increasing cultivation of nonnative cycads introduces opportunities for emerging pest dynamics (Whitaker *et al*., 2020). Invasive cycad pests such as the cycad scale (*Aulacaspis yasumatsui*) represent major threats to cycad conservation (Deloso *et al*., 2020; Marler *et al*., 2021), and even native herbivores may pose a threat in some circumstances: Recent host expansions have been documented among cycad‐feeding lepidoptera in places where native and nonnative cycads are co‐cultivated (e.g. botanic gardens), with potentially dire effects for *ex situ* cycad conservation (Marler *et al*., 2012; Normark *et al*., 2017; Whitaker *et al*., 2020).

## Coralloid root bacterial symbiosis

Early reports of an association between cycad roots and bacteria date from the late 19^th^ century with the anatomical description of morphologically distinct secondary roots, now termed coralloid roots (Fig. 2e,f; Schneider, 1894). Transverse sections of these roots will display a green ring‐like zone that is visible to the naked eye and often referred to as the cyanobacterial zone (Fig. 2f), due to the reported presence of *Nostocales* cyanobacteria (Grilli‐Caiola, 1980) that fix nitrogen inside the coralloid root in exchange for plant carbohydrates (Lindblad & Costa, 2002). Long thought to be the only symbiont present in the roots, recent research has shown that the cyanobacterial zone harbors a diverse taxonomic community (Zheng *et al*., 2018; Suárez‐Moo *et al*., 2019; Bell‐Doyon *et al*., 2020), although *Nostocales* remains the most abundant and best characterized cycad symbiont (Lindblad & Costa, 2002). The described symbiotic *Nostocales* cyanobacteria, or cyanobionts, are phylogenetically and taxonomically diverse and include species from the *Nostoc*, *Desmonostoc*, *Calothrix*, and *Aulosira* genera (Bustos‐Diaz *et al*., 2024; Cameron *et al*., 2024).

It is possible that the composition and/or function of the microbial communities is constrained by the soil conditions and may impact plant distribution. It has been reported in other systems that typical nitrogenases use molybdenum (Mo) as a cofactor (Bellenger *et al*., 2014); low soil availability of this element could require the use of alternative nitrogenases (Bellenger *et al*., 2020) that could be from either cyanobacterial (Nelson *et al*., 2019) or other bacterial origin (Harwood, 2020) and utilize vanadium (V) or iron (Fe) as a cofactor. The use of alternative nitrogenases could have a marked effect on the nitrogen fixation rates in the plant (Bellenger *et al*., 2014) and on the accuracy of previous estimates done in the field (Halliday & Pate, 1976). This has been proposed for *Encephalartos natalensis* (Ndlovu *et al*., 2023, 2024). It is also possible that, when present, fungal soil associates in the form of arbuscular mycorrhizal symbionts of noncoralloid roots (Fisher & Vovides, 2004) may help to enhance nitrogen fixation rates by promoting coralloid root development and increasing biomass, particularly in phosphorus‐limited soils (Fisher & Jayachandran, 2008). Biogeographically, coralloid root symbiotic associations can constrain or expand cycad species' habitat distribution. The distribution of N‐fixing plants, including cycads, has been suggested to be restricted by the dispersal limitations or enzymatic activity of their microbial symbionts, with soil conditions such as nutrient availability and pH affecting nitrogen fixation rate and species fitness (Bellenger *et al*., 2020; Delavaux *et al*., 2022).

**Fig. 3:**
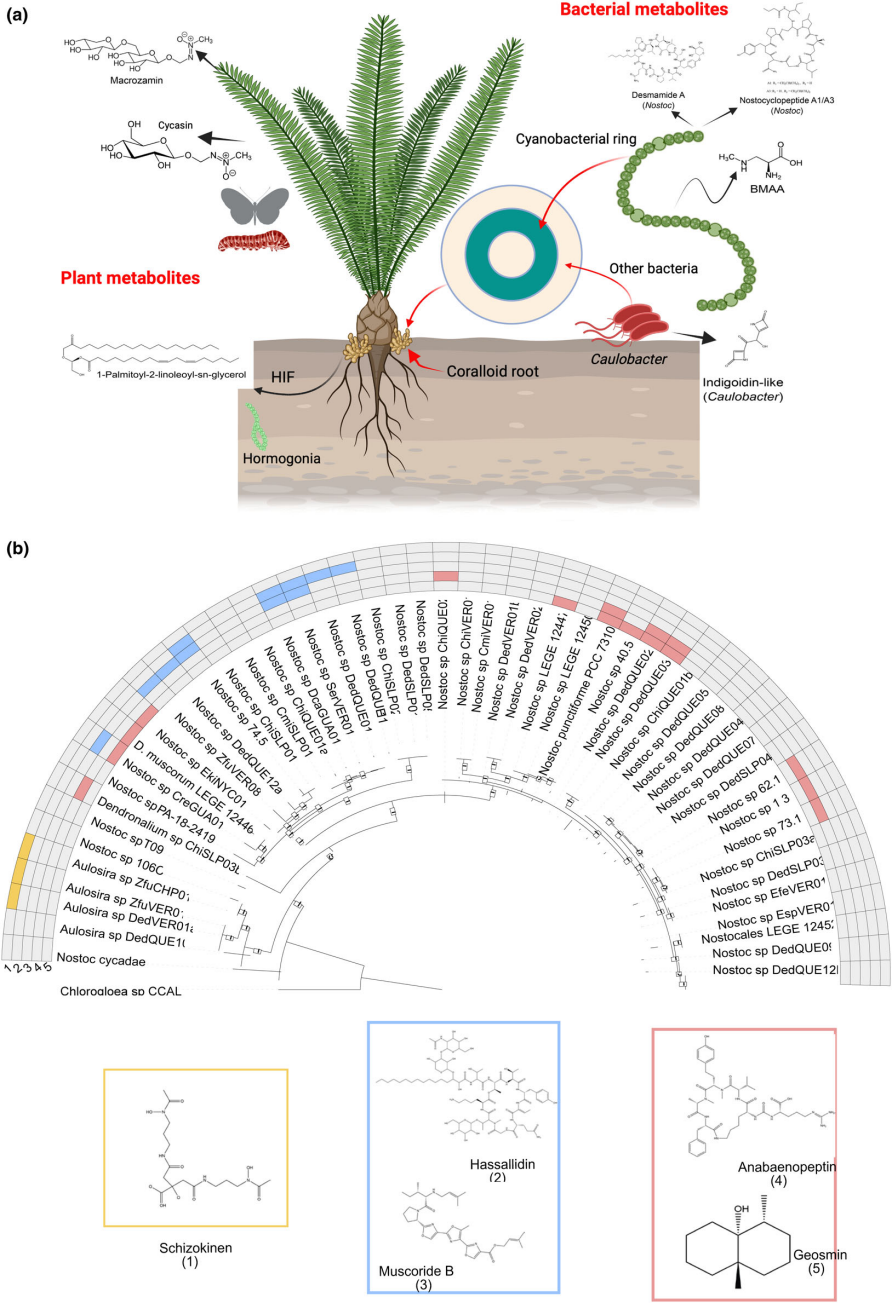
Known and predicted metabolites mediating cycad's symbioses. (a) Chemical structures of experimentally determined cycad‐derived specialized metabolites found in cycads and associated bacteria. Plant metabolites include the toxins macrozamin, cycasin (leaf and roots) and the hormogonium‐inducing factor (HIF) 1‐palmitoyl‐2‐linoleoyl‐sn‐glycerol produced in precoralloid roots to attract cyanobacteria. Symbiotic cyanobacteria produce β‐methylamino‐l‐alanine (BMAA), desmamide A, and nostocyclopeptide A1/A3, and associated *Caulobacter* produce indigoidine‐like metabolites, all within the coralloid roots. (b) Nostoc phylogeny of cycad cyanobionts with some of the biosynthetic gene clusters (BGCs) that they contain. The uneven distribution suggests that there may not be a universal metabolite associated with symbiosis. The phylogenetic distribution of BGCs is represented on the bacterial phylogeny by the matching color surrounding the chemical products of identified BGCs. (a) was created with Biorender.com (https://BioRender.com/c59s359). (b) was created using data from Bustos‐Diaz *et al*. (2024).

The establishment of symbiosis has been a recent area of research focusing on both bacterial and plant contributions. Conserved genes between all cycad cyanobionts have not been found, but experiments using *Nostoc punctiforme* Pasteur Culture Collection (PCC) 73102/American Type Culture Collection (ATCC) 29133 to infect other plants have found multiple genes crucial for the establishment of symbiosis at least in these strains (Wong & Meeks, 2002; Alvarez *et al*., 2022). This suggests that while specific traits are required for symbiosis, gene specificity, if any, is not apparent with the current data (Bustos‐Diaz *et al*., 2024). Moreover, most studies done to characterize these genes have neglected the noncyanobacteria microbes, limiting the possibility of identifying their role in symbiosis. From the plant side, signaling molecules produced by the precoralloid roots, such as diacylglycerols with hormogonia‐inducing factor activity, have been found to initiate plant‐symbiont recognition and trigger morphological changes in both the plant and the cyanobiont (Hashidoko *et al*., 2019; Fig. 3a). This cycad‐specific specialized metabolite incites the formation of motile filaments, or hormogonia, in susceptible *Nostocales*, which allows them to move toward the precoralloid root. While no cycad‐derived chemotactic metabolites have been experimentally characterized, the annotation of precoralloid root transcriptomes revealed the existence of terpenoids, which might play a role in the chemotactic attraction of the cyanobiont, along with multiple other genes (*RAD1*, *DHY*, *SymRK*, *EPP1*, V*APYRIN*, *CASTOR/POLLUX*, *NFP*, *CYTB561*, *GRAS*, and *HEP*) known to be involved in the establishment of legume‐rhizobial and plant‐mycorrhizal symbioses (Delaux & Schornack, 2021; Liu *et al*., 2022).

Chemical communication between the plant and the microbial community continues after colonization. Once inside the coralloid root, cyanobionts, and perhaps the rest of the microbiome, are subjected to metabolic manipulation by the cycad to bolster their nitrogen fixation rates (Lindblad & Costa, 2002) and produce nitrogenated amino acids, which are then transported to the rest of the plant (Pate *et al*., 1988). Among these is BMAA, a modified amino acid produced by the cyanobiont (Yan *et al*., 2020) that accumulates in the coralloid root (Marler *et al*., 2010) and is believed to play a role in cycads' defense against insect herbivores, though experimental evidence demonstrating insecticidal effects at naturally occurring doses is lacking (as mentioned in the previous section). The remainder of the metabolites or protein products involved in this interaction between host and symbionts remains uncharacterized. However, genomic analyses of cyanobiont strains have revealed that these genomes are enriched in biosynthetic gene clusters (BGCs), the bacterial operons that encode specialized metabolites (Freitas *et al.*, 2022; Bustos‐Diaz *et al*., 2024; Cameron *et al*., 2024). While the chemical products of most of the cyanobiont‐specific BGCs are currently unknown,phylogenetic analyses of the distribution of some previously described BGCs show that the presence of a specific BGC is mostly consistent with the phylogeny of these microorganisms (Fig. 3b), suggesting that members of each symbiotic clade may have a unique repertoire of specialized metabolites that may be involved with symbiosis. Thus, a key point for the future development of this research field would be to further characterize the chemical diversity of the cyanobionts to identify ecological ‘keystone metabolites’ mediating symbiosis. Moreover, future experiments should also take into account the existence of additional, yet largely ignored, metabolites synthesized by the accompanying microbiome that forms a syntrophic environment. So far, cyanobionts associated with *Caulobacter* species have been shown to produce a unique metabolite, an indigoidine‐like pigment, whose production is triggered when *Caulobacter* and their original cyanobiont are under nitrogen limitation conditions (Gutiérrez‐García *et al*., 2019). This suggests that members of the symbiotic community can, and likely do, interact among themselves (Teikari *et al*., 2025). The production of specialized metabolites could be an important characteristic of the accompanying bacteria that likely supports the symbiotic system through this and potentially other means (Liu *et al*., 2023; Ndlovu *et al*., 2023, 2024).

The ongoing and recent advances in the chemical ecology and genetics of root symbiosis described above have advanced our understanding of this complex system, yet there remain many necessary future directions of study. From a macroevolutionary standpoint, a recent study challenged the long‐standing hypothesis that coralloid roots are an ancestral trait in cycads and suggests instead that they convergently evolved in modern lineages (Kipp *et al*., 2024). This study opens further possibilities to explore potential local genomic and metabolic adaptations of the different cycad lineages, in particular those with contrasting habitats and biogeographic distributions. While great insights have been derived from the study of *Cycas* spp. and *Nostoc punctiforme* PCC 73102/ATCC 29133, wider exploration of cycad species and cyanobionts should be conducted. Furthermore, the characterization of the metabolic and taxonomic diversity of the greater microbiome is necessary to understand the molecular mechanisms that direct synthropic interactions between members of this bacterial community and the cyanobiont – and the effect that they might have on the cycad host.

## Cycad phyllosphere microbiota and interaction with the plant metabolome

Microbial communities, both bacterial and fungal, on and within plant tissues aboveground (the phyllosphere) are also likely impacting plant ecology and fitness. Phyllosphere microbiota was first explored in *Cycas panzhihuaensis* (Zheng & Gong, 2019), but their study did not include leaves, the most prominent cycad organ. The authors found that bacterial communities among reproductive organs (ovules, unfertilized and viable seeds) contain less bacterial diversity in comparison with roots that were highly similar and dominated by the family Enterobacteriaceae. Research on leaf fungal endophytes is limited to a few studies, that is, *Encephalartos* (Nesamari *et al*., 2017) and two *Zamia* species (Sierra *et al*., 2024; Villarreal Aguilar *et al*., 2024; Meléndez *et al*., 2025). The endophytic leaf fungi form a polyphyletic assemblage of mostly Ascomycota (80% of the amplicon sequence variants (ASVs)), while Basidiomycota, Mortierellomycota, and Mucoromycota were also present (Sierra *et al*., 2024). These initial diversity studies of leaf endo‐ and epiphytic microbes seem host‐specific and provide a veritable mine of data for further investigations into the ecological and evolutionary significance of the phyllosphere (Sierra *et al*., 2024). Two recent draft genomes from fungal endophytes isolated from *Zamia* spp. (*Neofusicoccum* sp. and *Xylaria* sp.) revealed a broad predicted repertoire of carbohydrate‐degrading Carbohydrate‐active Enzymes (CAZymes) (*n* = 450 and 446, respectively), peptidases (343 and 344), and predicted secondary metabolite clusters, with a significantly larger array in the *Xylaria* isolate (45 and 90) (Villarreal Aguilar *et al*., 2024). The fungal genomic features identified show the wide diversity of enzymatic and specialized metabolite clusters of endophytes, many of which are crucial for their colonization and host interactions favoring nutrient acquisition and production of toxins which may benefit the plant.

**Fig. 4:**
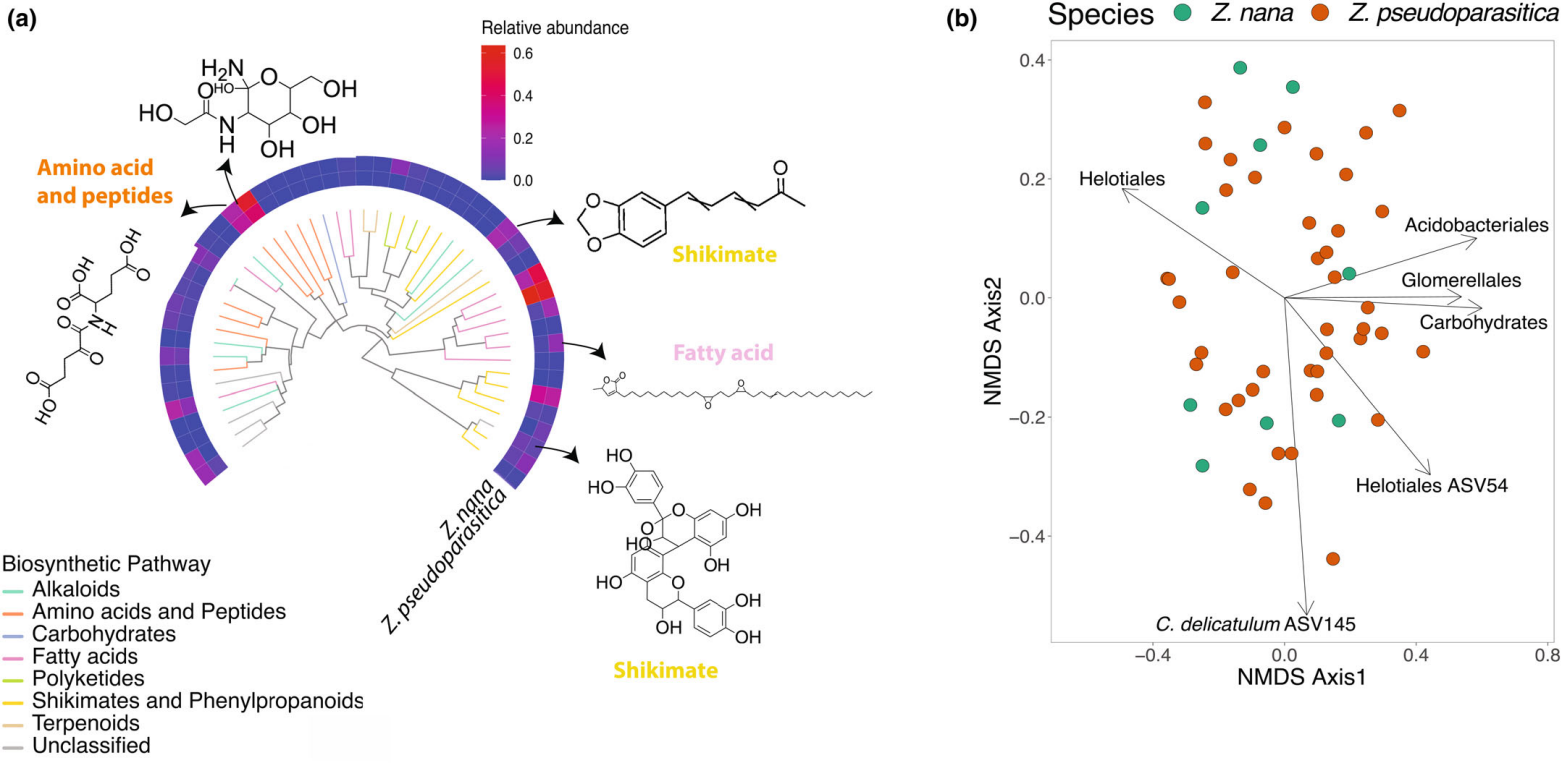
Leaf foliar metabolites differ between two ecologically isolated *Zamia* species. (a). A dendrogram using structural relatedness of foliar metabolites and the relative abundances of each leaf metabolite for the species *Zamia nana* (terrestrial) and *Zamia pseudoparasitica* (epiphytic). *Zamia* foliar metabolites are classified by their biosynthetic pathway as indicated by branch color (natural product classification). The chemical structure of five upregulated metabolites observed for *Z. pseudoparasitica* is presented. ClassyFire‐specific classification corresponds to *N*‐acyl‐alpha amino acids, glutamic acid (amino acid) and derivatives, benzodioxoles (shikimate), 1,2‐diacylglycerols (fatty acid), and biflavonoids and polyflavonoids (shikimate) (clockwise). (b). Foliar metabolome composition of *Z. nana* and *Z. pseudoparasitica* summarized into two dimensions with nonmetric multidimensional scaling (NMDS) based on pairwise chemical structural‐compositional similarity index, with arrows indicating the correlation (*P* = < 0.05) between the metabolome and the microbiome. Both figures modified from Sierra *et al*. (2024) with permission of Springer (License 5985531162609).

Cycads are known for harboring a diverse range of anti‐herbivore and toxic compounds, whose origin and impact across trophic levels have been a source of great debate (Marler *et al*., 2010 and references therein). A comprehensive review of cycad phytochemicals emphasizes the distinct chemical profiles of most genera and the potential source of specific pharmacological and toxicological properties (Du *et al*., 2024). Sierra *et al*. (2024) studied the association of plant‐microbiota (bacteria and fungi) with the intraspecific leaf metabolome composition of two sympatric species (*Zamia nana* and *Z. pseudoparasitica*) to address local adaptive responses (terrestrial vs epiphytic species). Among the 49 abundant metabolites, five compounds (benzodioxoles, biflavonoids and polyflavonoids, 1,2‐diacylglycerols, N‐acyl‐alpha amino acids, and glutamic acid and derivatives) were more expressed in *Z. pseudoparasitica* compared to *Z. nana* (Sierra *et al*., 2024) (Fig. 4). The metabolite variation was correlated to bacterial and fungal leaf endophyte community composition and significantly associated with the abundance of the bacterial order Acidobacteriales and fungal orders Helotiales and Glomerellales. Specific fungal taxa classified as Heliotales ASV54 and *Cladosporium delicatulum* ASV145 were strongly associated with *Zamia* foliar metabolites along the NMDS Axis 2. It is possible that such an association speaks only of local adaptive responses of these two species to their habitat, as found in *Dioon* (Gutiérrez‐García *et al*., 2019). We are just beginning to unravel the association of cycad metabolites with phyllosphere microbiota and the ecological consequences for associated insects. Defensive secondary metabolites in cycads have clear impacts on plant fitness and are likely influenced by complex plant–microbe reciprocal interactions. Future research should work to further elucidate the taxonomic and biochemical diversity of the cycad phyllosphere and describe any co‐interaction between host and endophytic microbes.

## Conclusion

Here, we have reviewed the last decade's worth of research on cycad chemical ecology and symbioses. These exciting recent discoveries position cycads as integral to our understanding of seed plant evolution and ecology, with far‐reaching implications for the biological sciences. Cycads' diverse associations with insects have directed new attention to a broader range of signaling methodologies and provided fertile ground for investigations into adaptation and coevolution. Studies on coralloid root symbioses have highlighted the importance of multispecies bacterial communities in cycad–cyanobacteria symbiosis and opened the door for studies on the ecology of specialized metabolites. Similarly, investigations of plant phyllosphere microbiota have identified previously overlooked putative symbionts and potential sources of metabolic protection from herbivores. Overall, cycads and their biotic associates provide a rich study system for investigating the chemistry of ancient co‐evolution and microevolutionary and ecological interactions.

Much of the current research on cycads can be unified through the lens of chemical ecology, as we become increasingly aware of the myriad ways in which chemodiversity drives interactions with other organisms. As an early diverging lineage of seed plants, investigations into cycad chemical trait evolution and ecology are particularly well placed to shed light on the origins and maintenance of plant and microbial chemodiversity over long timescales and the implications for mutualistic and parasitic insect associations. Further investigations into cycad metabolic diversity and the ecology of microbiomes will improve our understanding of the mechanisms underlying plant–microbe symbioses across all land plants. The recent advances in our understanding of the chemical ecology of cycad symbiosis have unlocked a new interpretation of the Rosetta Stone metaphor and lay the groundwork for furthering our understanding of the ecology and evolution of species interactions from the macro to the micro across all of Plantae. Whether at the metaorganism or holobiont scale, our continued investigations into the mechanisms underlying cycad symbioses have great implications for our understanding of ecological functioning, lineage persistence, and resilience to climate change. These plants have persisted across evolutionary time through a host of chemically mediated symbiotic associations and, as Knut Norstog first noted, provide a bridge between extant and early seed plants (Donaldson, 2003). As such, they help scientists to connect diverse research disciplines and continue to provide insights into the evolution and ecology of seed plants.

## Competing interests

None declared.

## Author contributions

JCVA conceived the idea of the review and wrote the phyllosphere along with AMS. The coralloid root section was written by EDB‐D, AC‐J, JCVA and FB‐G. MRLW wrote the herbivory section, and SS wrote the pollination section and remaining sections and edited the manuscript.

## Disclaimer

The New Phytologist Foundation remains neutral with regard to jurisdictional claims in maps and in any institutional affiliations.
